# Unravelling how and why the Antiretroviral Adherence Club Intervention works (or not) in a public health facility: A realist explanatory theory-building case study

**DOI:** 10.1371/journal.pone.0210565

**Published:** 2019-01-16

**Authors:** Ferdinand C. Mukumbang, Brian van Wyk, Sara Van Belle, Bruno Marchal

**Affiliations:** 1 School of Public Health, University of the Western Cape, Cape Town, South Africa; 2 Department of Public Health, Institute of Tropical Medicine, Antwerp, Belgium; McGill University Faculty of Medicine, CANADA

## Abstract

**Background:**

Although empirical evidence suggests that the adherence club model is more effective in retaining people living with HIV in antiretroviral treatment care and sustaining medication adherence compared to standard clinic care, it is poorly understood exactly how and why this works. In this paper, we examined and made explicit how, why and for whom the adherence club model works at a public health facility in South Africa.

**Methods:**

We applied an explanatory theory-building case study approach to examine the validity of an initial programme theory developed *a priori*. We collected data using a retrospective cohort quantitative design to describe the suppressive adherence and retention in care behaviours of patients on ART using Kaplan-Meier methods. In conjunction, we employed an explanatory qualitative study design using non-participant observations and realist interviews to gain insights into the important mechanisms activated by the adherence club intervention and the relevant contextual conditions that trigger the different mechanisms to cause the observed behaviours. We applied the retroduction logic to configure the intervention-context-actor-mechanism-outcome map to formulate generative theories.

**Results:**

A modified programme theory involving targeted care for clinically stable adult patients (18 years+) receiving antiretroviral therapy was obtained. Targeted care involved receiving quick, uninterrupted supply of antiretroviral medication (with reduced clinic visit frequencies), health talks and counselling, immediate access to a clinician when required and guided by club rules and regulations within the context of adequate resources, and convenient (size and position) space and proper preparation by the club team. When grouped for targeted care, patients feel nudged, their self-efficacy is improved and they become motivated to adhere to their medication and remain in continuous care.

**Conclusion:**

This finding has implications for understanding *how*, *why* and under *what* health system conditions the adherence club intervention works to improve its rollout in other contexts.

## Introduction

The number of people living with HIV/AIDS (PLWHA) in South Africa has reached an estimated 7.9 million [1]. Keeping in line with the ‘90-90-90’ goal (90% of all PLWHA knowing their HIV status, 90% of all people diagnosed with HIV infection receiving sustained antiretroviral therapy (ART), and 90% of all people receiving ART achieving viral suppression) by 2020 [2], South Africa has established various policies, programmes and strategies.

To increase the number of people knowing their status, various voluntary counselling and testing campaigns are organised nationwide. To improve the number of PLWHA on sustained ART, the South African government has adopted the WHO’s ‘test and treat’ treatment guidelines of 2015 as of September 2016 [3]. Enhancing the number of PLWHA suppressed virally entails retaining them under the care umbrella and encouraging them to adhere to their treatment. To this end, in addition to using the primary health-care facilities to drive the HIV-treatment and care programme, various differentiated care models (tailored care packages to suit the needs of different patient groups) of the ART have been designed and implemented.

The adherence club is one such differentiated care models that was implemented in the Western Cape Province of South Africa. The model, which is health-care worker-managed [4], was designed to shift most consultations and ART collections for stable patients into organised group ART care. The adherence club intervention has been described previously [5–7]. The adherence club programme aims to (a) retain patients in ART care by providing a more efficient way to manage stable patients; (b) maintain good long-term adherence in PLWHA on ART through quick access to medication and (c) decongest the health facility through group sessions that are facilitated by trained non-clinical health-care workers [3]. The adherence club intervention has shown better effectiveness of improving and sustaining retention in care and adherence to medication compared to the standard care services reported in four studies [8–11].

Based on the evidence supporting the effectiveness of the adherence club intervention [8–11] and its cost-effectiveness [12], there are calls to adopt this differentiated treatment and care model for the management of HIV [13]. Indeed, the WHO’s 2015 consolidated treatment guidelines for HIV recommend the use of group-based ART models to improve retention in care and enhance treatment adherence [14]. Despite these calls, the literature provides little or no theory-based understanding of how and why these interventions work [15].

Van Belle and colleagues [16] argue for more use of theories in implementation studies. The authors proposed that theory-driven approaches, such as realist evaluation, have the potential to demonstrate the complex interplay between a programme (and an intervention), context, targeted actors, causal mechanisms and expected outcomes. In this paper, we examined and made explicit what aspects of the adherence club intervention work, for what patient population and under which contexts using a theory-driven approach, realist evaluation. We described the process of testing the initial programme theory of the adherence club intervention in a public health clinic in the Western Sub-District of the Western Cape Province, South Africa. This is important for clarifying the programme theory that underlines the adherence club intervention.

## Methodological approach

**Fig 1:**
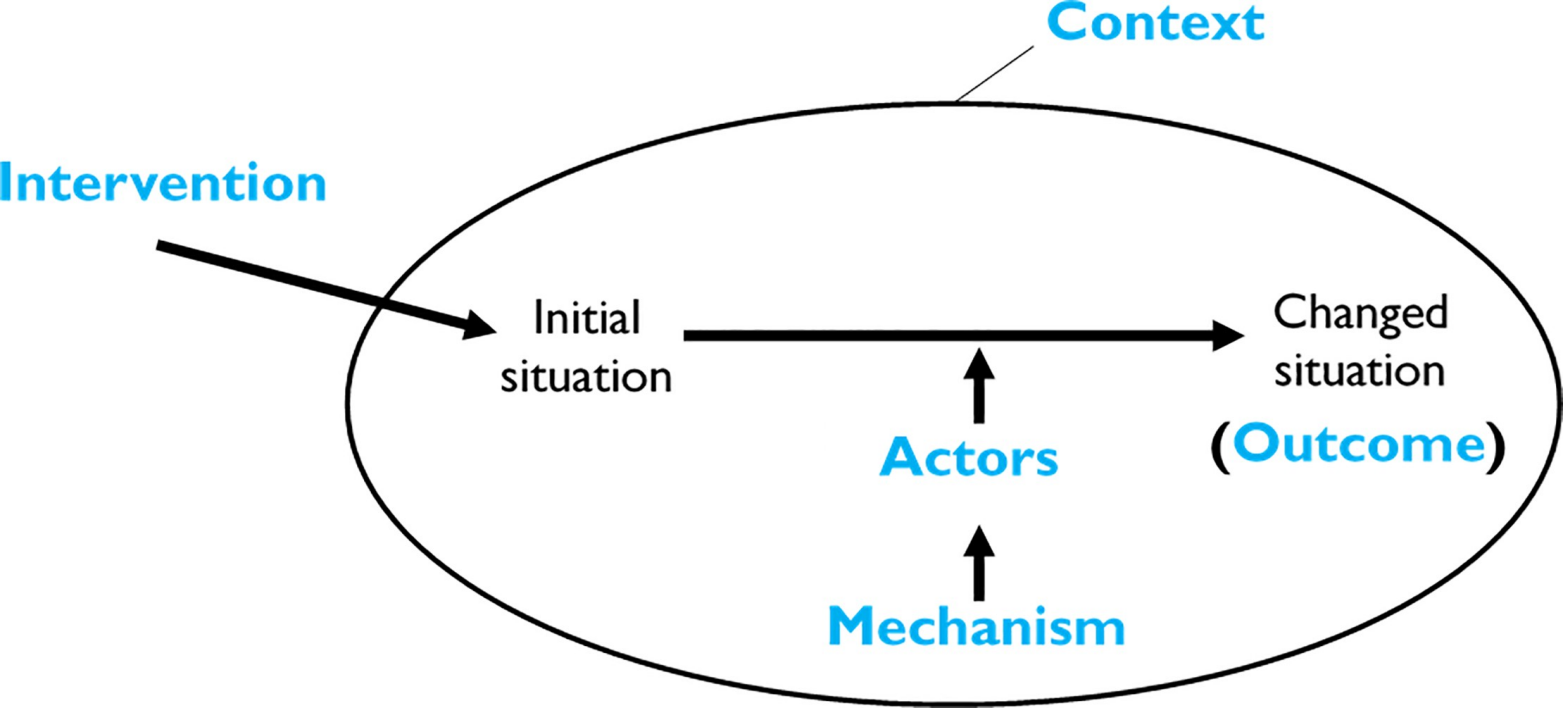
The ICAMO configuration.

Realist evaluation is a theory-driven evaluation approach drawn from the seminal work of Pawson and Tilley [17]. Realist evaluation starts by clarifying the 'programme theory'–the set of assumptions of programme designers (or other actors involved) that explain how they expect the intervention to achieve its objective(s). The realist evaluator hypothesises the intervention, the relevant actors through whom the intervention is expected to work, the mechanisms that are likely to operate, the contexts in which the mechanisms might operate and the anticipated outcomes [18]. This process is known as constructing the intervention-context-actor-mechanism-outcome (ICAMO) hypotheses [19,20] (**Fig 1**).

Realist evaluation is about theory-testing and refinement [21], whereby the evaluator assesses whether a programme is designed in such a way that it can achieve its intended outcomes and how it does so [22]. The initial programme theory guides the assessment of the effectiveness of the intervention and the consistency of the implementation [23]. The outcome of this process is a ‘generative theory’ of causation, whereby there is an interaction between the actors and the components of the intervention leading to a transformation (outcome) through causal powers (reasoning of the actors) [24].

Realist analysis starts with the understanding that the programme under evaluation will have varying implementation outcomes [18]. While implementation scientists suggest that this variation in outcome could be attributed to the variation in the context in which the intervention is implemented [25], for realists, the outcome variations are attributed to variations in the ICAMO as a configuration and not only the context. Realist evaluators go into the evaluation process with some expectations guided by the initial programme theory. During the evaluation, some expectations are confirmed, some might prove misguided, and the end product of the analysis is expected to improve the picture of the programme efficacy and inefficacy [26].

In our previous work, we formulated the initial programme theory of the adherence club intervention [27]. We conducted an exploratory qualitative study of programme designers’ and managers’ assumptions and perspectives of the intervention and carried out a document review of the design, rollout, implementation and outcome of the adherence clubs [28]. We also conducted a systematic review of available studies on group-based ART adherence support models in sub-Saharan Africa to tease out their underlining theories [29]. In addition, we carried out a scoping review of social, cognitive and behavioural theories that have been applied to explain adherence to ART [30]. Using the process of configuration mapping, we constructed an ICAMO map representing the initial programme theory of the adherence club, through the process of retroduction–mechanism centred logic and analysis [27]. Finally, we used the "if…then…because" statements to translate the ICAMO configuration map into a testable hypothesis (**Box 1**) [31].

**Box 1. Initial programme theories of the adherence club intervention represented by two tentative theories (hypotheses)**

**Initial programme theory 1**

**IF** adult (18+ years) clinically ‘stable’ patients with evidence of good clinic attendance are group-managed, receive quick symptom checks, quick access to medication, consistent counselling and social support from the peer counsellor,

**THEN** patients are likely to adhere to medication and remain in care,

**BECAUSE** they develop a group identity, which improves their perceived social, support, satisfaction and trust; and acquire knowledge, which helps them to understand their perceived threat and perceived benefits and improves their self-efficacy. As a result, they become encouraged, empowered and motivated, thus, more likely to remain in care and adhere to the treatment.

**Initial programme theory II**

**IF** operational staff receive goals and targets set to continuously enrol patients in the adherence club and strictly monitor their participation through strict standard operating practices (the promise of exclusion in the event of missed appointment and active patient tracing),

**THEN** patients are likely to adhere to medication and remain in care,

**BECAUSE** they fear (perceived fear) of losing the benefits (easy access to medication, peer support, reduced waiting times, and two-month ART collection) of the club system and they are coerced through adhesive club rules. As a result, they become nudged to remain in care and adhere to the treatment, which might decongest the health facility.

The goal of testing the initial programme theory of the adherence club intervention (an evaluation of the adherence club intervention) is to verify, refute and/or modify it. In this paper, we sought to unravel the mechanisms explicating how, why, for whom and in what circumstance the adherence club programme works at a community health centre in Cape Town.

## Research design

Within a realist evaluation, we applied an explanatory theory-building case study approach to testing the initial programme theory with the goal of refuting, validating or refining it. We adopted the multiple embedded case study design [32] with Facility X being one of the cases. Facility X was considered is a unit of analysis, with each of its adherence clubs being sub-units. Theory-testing using case studies evaluates the explanatory power of theories and their boundaries [33] so as to develop context-specific causal explanations of how and why programmes work [32]. In realist logic, mechanisms–the process of how subjects interpret and act upon the intervention (or components of the intervention)–are at the heart of causal explanations [34] and the case study approach provides a platform to illuminate mechanisms in relation to outcomes.

The case study approach is appropriate in teasing out the mechanisms related to a programme or an intervention because it allows movement from the emic (the perspective of the subject) to etic (perspective of the researcher) accounts of a phenomenon [33]. It also allows for the use of multiple methods of data collection [18], which favours iterative investigation (repeated movement between data analysis and collection) and recognises the importance of context [6]. These characteristics make the case study approach methodologically complementary to realist evaluation, thus providing a “close-in” on real-life situations to test the theory under investigation [6].

**Fig 2:**
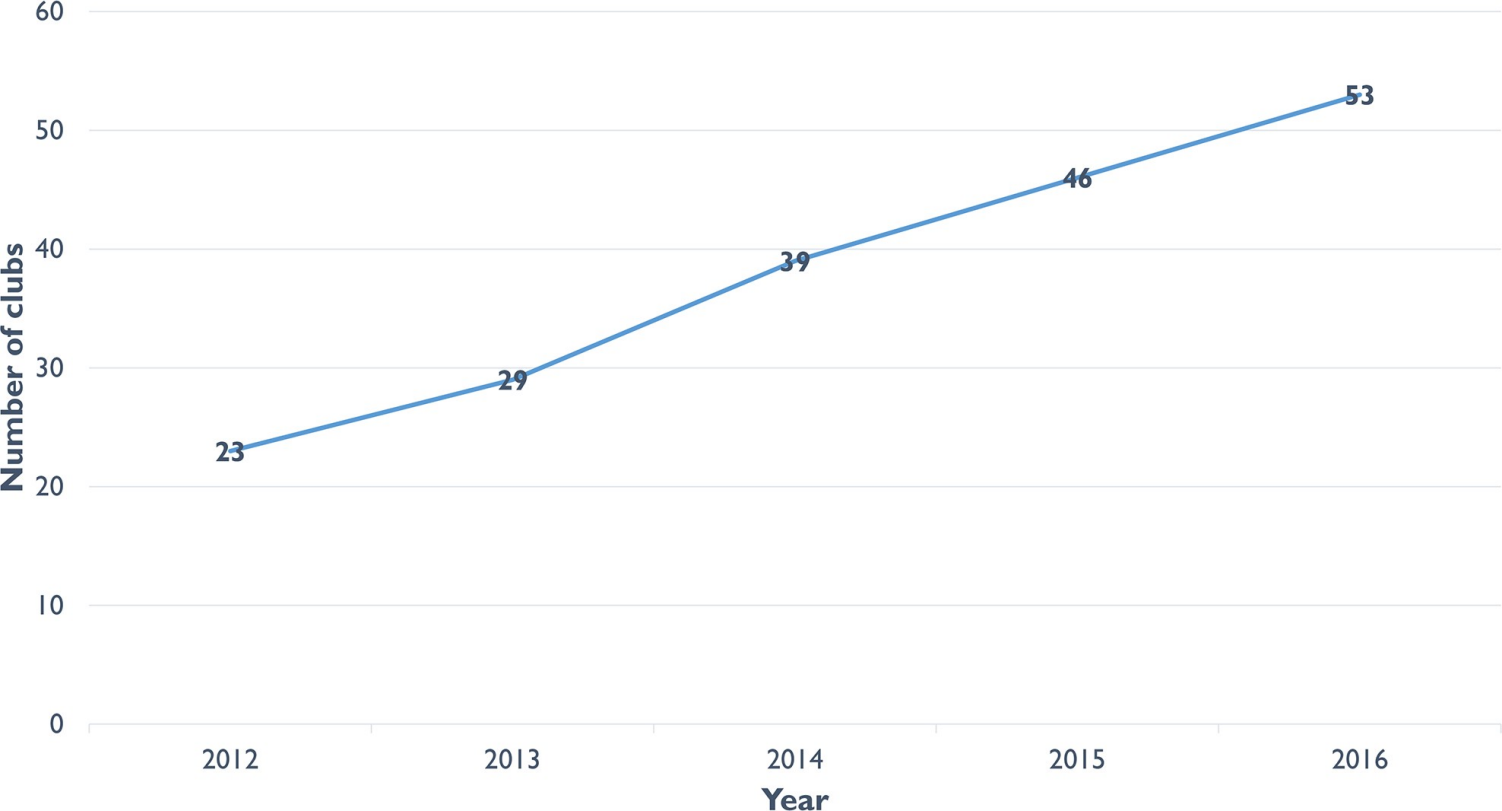
Number of adherence clubs, which opened from 2012 to 2016 at Facility X.

Cases in case study research are described as typical, deviant or crucial [35]. Facility X, where the initial programme theory was first tested, represents a ‘typical’ example of an adherence club in terms of the rollout of the adherence club programme because Facility X was among the facilities where the rollout was initiated in 2012. Since its inception, the adherence club programme in Facility X has shown steady growth at a reasonable pace (**Fig 2**).

## Study setting

Mitchell’s Plain is one of Cape Town's largest townships established on the Cape Flats about 20 miles from the city centre in the mid-seventies as a ‘dormitory suburb’ for ‘Coloured’ people. The Mitchell’s plain township has about 290,000 inhabitants and comprises several sub-sections reflecting the diverse class backgrounds of the population.

Facility X is a primary health-care facility that provides free first-level primary and some second-level health services to the Mitchells Plain community and surrounding areas. Facility X operates a 24-hour trauma unit for emergencies, maternity and obstetric unit, mental health services, specialised paediatric services for children up to five years old, an outpatient department, pharmacy and dispensary, an antiretroviral clinic, and a chronic diseases of lifestyle (CDL) clinic (including CDL clubs) [36].

Facility X is an accredited ART initiation and on-going management site, which provides HIV counselling and testing (HCT) and associated support services. Facility X was selected as one of the ART sites for the first phase rollout of the adherence club programme and officially formed its first adherence club in March 2012. Based on the routine monitoring data of the adherence club programme, an estimated 3,600 patients were receiving ART care at Facility X by the end of 2016 and of these, 1,368 adult patients were retained in care through the adherence club. The routine data also show that 53 adherence clubs had been created by the end of 2016 at Facility X. In **Fig 2**, the number of clubs that were established each year since the inception of the adherence club programme in 2012 is shown.

## Methods

The realist evaluation approach is method neutral, i.e. quantitative and qualitative data are collected as part of the programme theory that is being ‘tested’. The use of a multi-method evidence base to ensure good documentation of the implementation of the programme is encouraged [37]. To this end, we collected and analysed quantitative and qualitative data.

**Fig 3:**
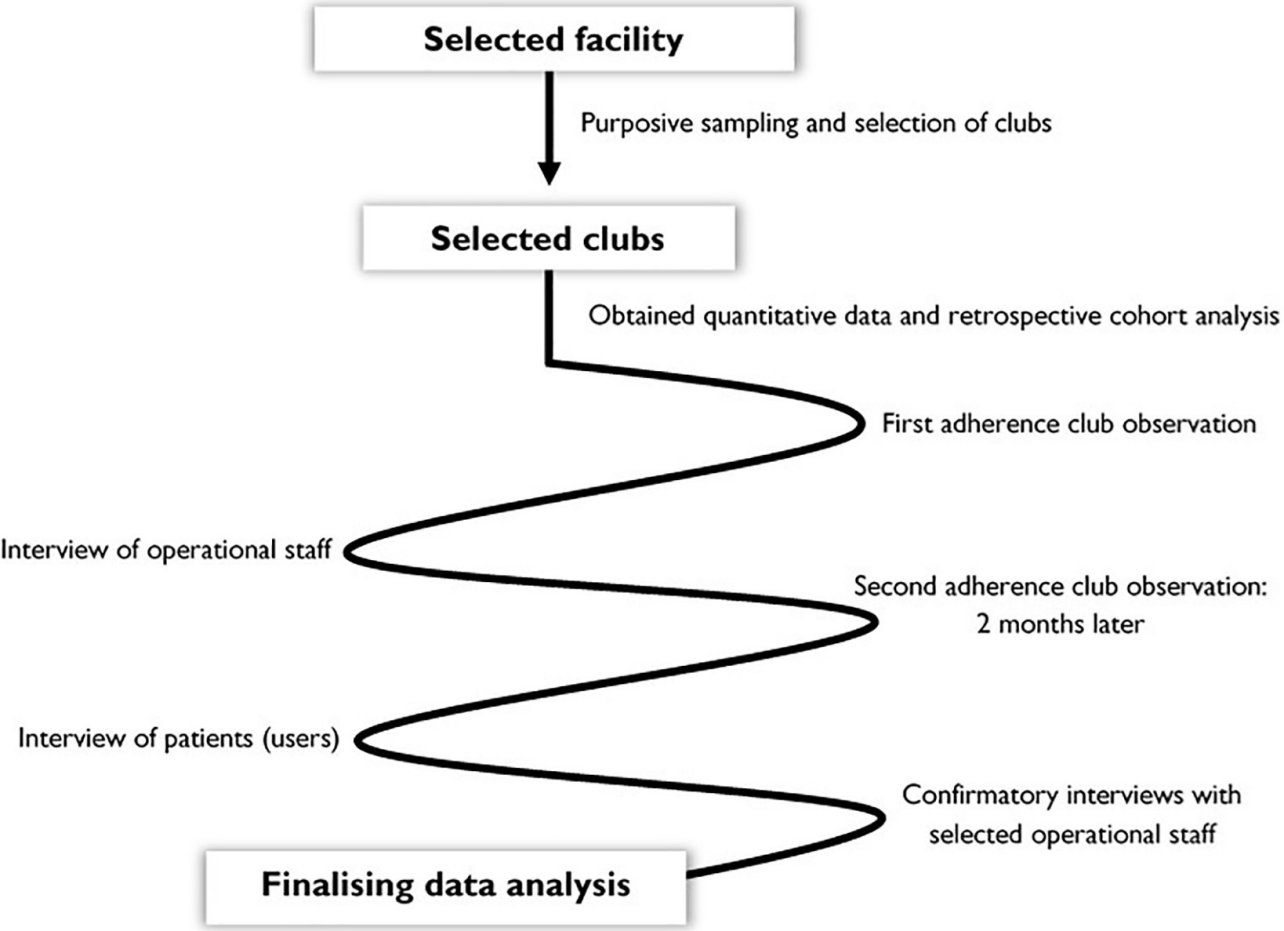
Data collection approach/strategy.

In our study, we used quantitative data to identify and classify the outcome patterns and qualitative data to explore implementation features related to the context (observation) and the mechanism (in-depth interviews). We thus combined a retrospective cohort analysis and an explanatory qualitative approach. The retrospective cohort analysis was conducted to describe the primary outcomes of the adherence club intervention (retention in care and adherence to medication) and the qualitative explanatory design provided evidence regarding the ICAMO configuration links in the implementation chain (intervention modalities, actors involved, generative mechanisms, relevant context and outcome patterns). **Fig 3** is an outline of the data collection strategy that was adopted.

Regarding the retrospective cohort study, data were obtained from the adherence club registers from the facility. The adherence club register records details of the retention in care behaviour through club attendance and of adherence through the recoded viral load results. In **Table 1** the different modalities related to retention in care as recorded in the adherence club register are shown [38].

**Table 1:** Codes for the recording of retention in care outcomes in the club register.

Recorded Outcome	Outcome Event
DNA	**Did not attend** club session and did not come to the clinic or send a buddy within five days after the club sessions
BTC	**Back to Clinic**- Exiting the club for medical reasons and reinstated in the routine standard of care
TFOC	**Transferred out to a different club**–Patient is transferred out to another club in the same facility
TFO	**Transfer out**–Patient is leaving the facility completely and will attend a clinic elsewhere
RIP	**Rest in Peace–**Patient has died

The outcome of adherence is identified through the recoded values of the viral load measurements taken six-monthly.

Two qualitative data collection methods were used: non-participant observations [39,40] and semi-structured realist interviews [41,42]. Non-participant observations allowed us to gain insights into the various types of club sessions, different activities and the dynamics of interactions between the patients with each other and with the health-care providers. We used an observation guide that details the interactions, processes, or behaviours to be observed during the club sessions (**Additional file 1**).

The second qualitative method we applied was the semi-structured realist interview [43], whereby the initial programme theory is placed before the interviewee to comment with the view of providing refinement. In this way, ‘*the researcher's theory is the subject matter of the interview*, *and the subject is there to confirm or falsify and*, *above all*, *to refine that theory*’ [41]. In addition to commenting on our theories during the interview process, we focused on capturing the participants’ perspectives and experiences of the programme [42]. **Additional files 2 and 3** present the questions tailored for the different groups of study participants.

### Sampling approaches

Regarding the quantitative study, we purposively selected two clubs among the 23 that were opened and reached the maximum capacity (35–45 patients) in 2012. First, we identified the clubs that had reached the maximum capacity. Of the 23 clubs that opened in 2012, eight reached the maximum capacity. Then, from the eight identified clubs, we used the fishbowl or lottery method of sampling [44] to obtain two adherence clubs to be included in the analysis. Club A had 47 patients and Club B had 39 patients.

The selection of the interviewees was based on their potential contributions toward clarifying the initial programme theory [45]. To this end, we adopted the purposive sampling technique. Pawson and Tilley suggested that practitioners have specific ideas on what it is within the programme that works (M), knowledge on the outcomes (O) of the programme (because they are likely to have experienced successes and failures) and some awareness of people and places (C) for whom the programme works [41,45]. In their view, the beneficiaries are more likely to provide relevant information related to the mechanisms (M) than to its contextual constraints (C) and outcome patterns (O).

In **Table 2** the different groups of participants who were interviewed are outlined. Following Pawson and Tilley’s insights, we interviewed 12 participants–three categories of actors: clinical staff (doctors and nurses), club facilitators (lay counselors) and the users (patients). These different groups of actors were purposively selected based on their knowledge of the adherence club intervention.

**Table 2:** Distribution of study participants.

Stakeholder	Number of participants	Time on Adherence club
Doctors	1	Since 2012
Nurses	2	Nurse 1–2012 Nurse 2–2010
Counsellors (Club facilitators)	2	Counsellor 1–2012 Counsellor 2–2012
Patients (club members)	5	Patient 1–2017 Patient 2–2012 Patient 3–2010 Patient 4–2014 Patient 5–2015
Patients (former club members)	2	Ex-member 1–2014 Ex-member 2–2015

## Ethical considerations

This study is part of a larger project “A realist evaluation of the antiretroviral treatment adherence club programme in selected primary health-care facilities in the metropolitan area of Western Cape Province, South Africa”, which has received ethics clearance from the University of the Western Cape Research Ethics Committee (UWC REC) (Registration No: 15/6/28). In addition, we obtained ethical clearance from the Provincial Department of Health of the Western Cape Province. Furthermore, we obtained the permission of the facility head and management before data collection processes commenced.

At the level of the study participants, we first provided the interviewed participants with an information sheet of the project. This was followed by a verbal explanation of the role of the participant and the significance of their participation. They were required to sign an informed consent form. We promised and ensured confidentiality and anonymity by identifying the participants using pseudo names and by password-protecting all files related to the study.

## Data analysis

The realist analysis process “*is an ongoing iterative process of placing nuggets of information within a wider configurational explanation*” [42]. The data analysis proceeded in two steps: (1) separate analysis of the quantitative and qualitative components of the data and (2) synthesis of the findings from the quantitative and qualitative arms through configurational mapping using the ICAMO heuristic tool.

The quantitative data were analysed using the Kaplan-Meier descriptor survival analysis approach [46]. The Kaplan-Meier method is a nonparametric method used to estimate the probability of survival past given time points and thus calculates a survival distribution. The goal of this analysis was to describe the rate at which patients enrolled in the adherence club at the facility, became lost to follow-up, and sent back to clinic care or died–proxies to retention in care. We aimed at describing the conditional probability of a patient remaining in club care at the end of 6, 12, 24 and 36-month intervals.

Because ART adherence is a behaviour that can be linked to a biological marker (HIV-1 viral load), we used the Kapan-Meier descriptor to describe the rate at which a viral load rebound occurred in the patients. This follows the premise that all patients admitted into the adherence club should have viral loads reading at “lower than detectable (LDL)”.

Data from the field notes and interviews were analysed using the thematic content analysis method [47] and classified according to aspects of the intervention, actors, context, mechanisms and outcomes–components of the ICAMO heuristic tool. To this end, codes related to components of the initial programme theory were allocated to chunks of the text [48], a process described as deductive thematic analysis [49]. This deductive thematic analysis, which is distinct from typical inductive qualitative analysis, was appropriate because the ICAMO framework was applied to the data at the outset. During the analysis, emergent codes were added to the coding tree, which was periodically revised. Through this process, we obtained conjectured ICAMO configurations, which were confirmed through the application of counterfactual thinking (testing possible alternative explanations).

## Results

We first discuss the findings of the quantitative retrospective analysis, then the thematic analysis of the interviews and observation notes.

## Quantitative findings

The retrospective descriptive analysis was used to describe the retention in care and adherence behaviours (principal outcomes) of the patients in the ART club. We used the Kaplan-Meier method [46] to describe the rate at which patients dropped out of club care (retention in care) and failed to maintained viral loads lower than detectable (≤400 copies/mL).

### Retention in care

The total number of patients retained in club care (81.4%) after 36 months in care in the two sampled adherence Clubs A and B are shown in **Table 3**. Club A has an overall retention rate of 78.7% and Club B has a retention rate of 84.6%.

**Table 3:** Percentage of patients receiving care in the two adherence clubs retained in care after three years.

Adherence Club	Total Number	Number patients LTFU	Remaining in care	
			Number	Percent
**Club A**	47	10	37	78.7%
**Club B**	39	6	33	84.6%
**Overall**	86	16	70	81.4%

**Fig 4:**
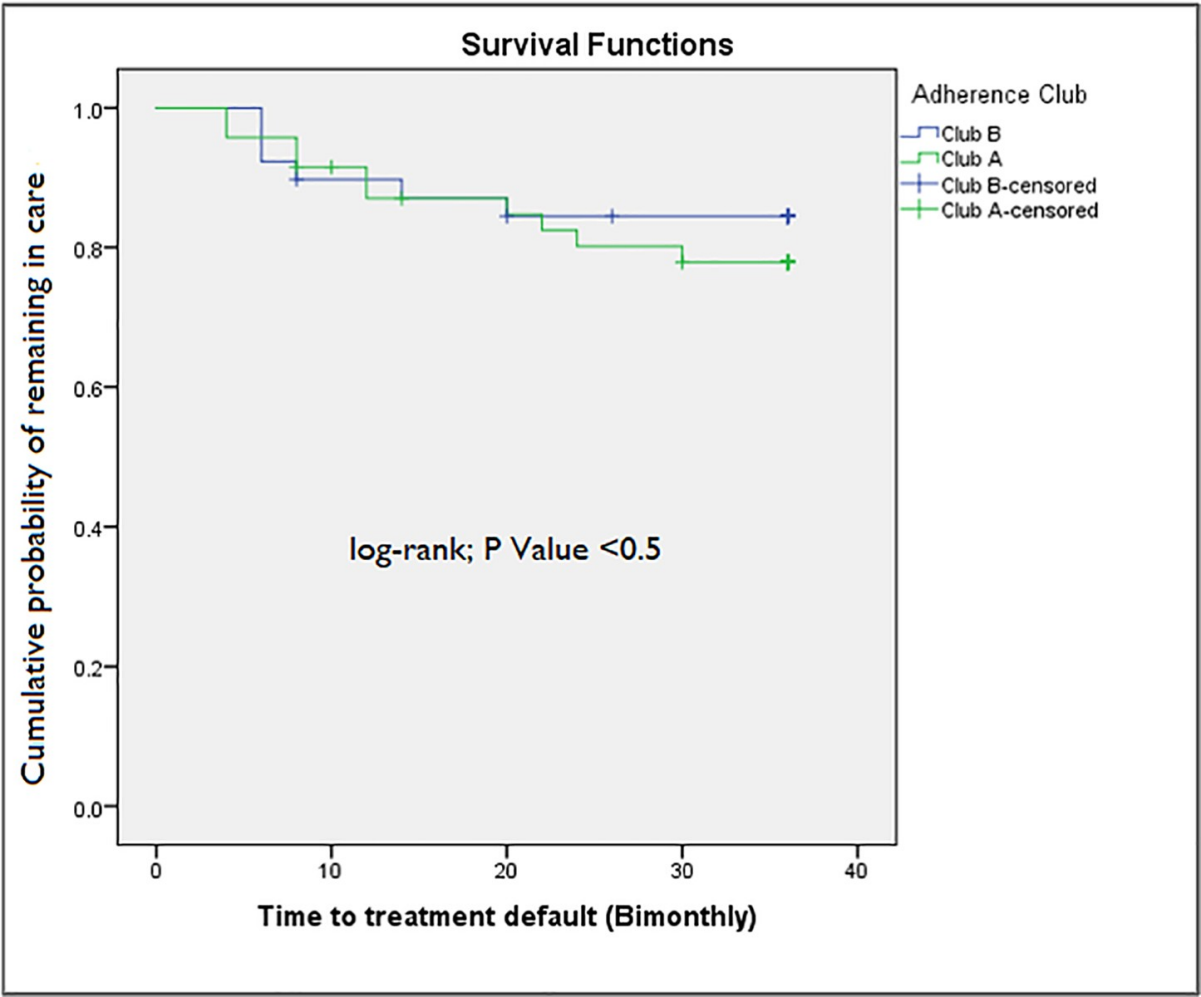
Cumulative probability of patients remaining in care in adherence Clubs A and B.

In **Fig 4**, the survival (retention in care) distributions of the patients receiving care in the two adherence clubs are shown to help us understand how the survival distributions compare between these two clubs. The frequent ‘interactions’ among the survival distributions indicate little differences between them.

Apart from describing the overall retention in care rates of the patients receiving care in the adherence clubs, we also investigated the retention in care rates at various intervals. In **Table 4** the probability that a patient receiving ART in adherence Clubs A and B will be retained in care at six, 12, 24, and 36 months are displayed.

**Table 4:** Kaplan-Meier estimates of the retention in care behaviours of patients attending two adherence clubs at Facility X.

Duration of follow-up	Remaining in care Club A %(95% CI)	Remaining in care Club B %(95% CI)
**6 months**	95.7% (84.2–99.2)	92.3% (78.0–97.9)
**12 months**	87.0% (73.3–94.5)	87.1% (71.6–95.1)
**24 months**	80.1% (65.5–89.9)	84.6% (68.6–93.4)
**36 months**	78.7% (62.9–88.2	84.6% (68.6–93.4)

We further conducted a log-rank test (Mantel-Cox) to determine whether the survival distributions of the two adherence clubs are statistically significantly different. If significance (*p-value*) is defined as p<0.05, then we can conclude that the survival distributions of the two adherence clubs are very similar. This suggests that there is a level of consistency regarding the capacity of the adherence club intervention to retain patients in care at Facility X.

### Adherence to medication (suppressive adherence)

Viral load is used as a proxy for adherence to antiretroviral medication. In the adherence club, viral loads are measured once a year. Our analysis considered three viral load measurements within the three-year period of the study. Using the virological failure indicator (>400 cells/cm^3^) to define the outcome of interest, an overall population adherence level of 95% was achieved within the three years of the study at the adherence Clubs A and B. The adherence breakdown of the two adherence clubs are shown in **Table 5**.

**Table 5:** Population level adherence rates of two adherence clubs in Facility X.

Adherence Club	Total Number	Non-suppressive adhering patients	Adhering patients	
			Number	Percent
**Club A**	47	3	44	93.6%
**Club B**	39	2	37	94.9%
**Overall**	86	5	82	94.2%

**Fig 2** and **Table 6** indicate that the adherence to medication of patients in the adherence club is good. After receiving treatment for six months, the adherence behaviour assessed by viral load of the patients was 100% at both sampled clubs. At 36 months, the adherence rates of Club A were 93.6% and of Club B 94.9%. These represent very good population-level adherence levels.

**Table 6:** Suppressive adherence rates at various intervals of the two sampled adherence clubs.

Duration of follow-up	Adherence Club A % (95% CI)	Adherence Club B % (95% CI)
**6 months**	100.0% (90.5–100)	100.0% (88.8–100)
**12 months**	95.7% (87.1–99.8)	100.0% (88.8–100)
**24 months**	92.9% (84.1–98.2)	94.9% (79.8–98.6)
**36 months**	92.9% (84.1–98.2)	94.9% (79.8–98.6)

**Fig 5:**
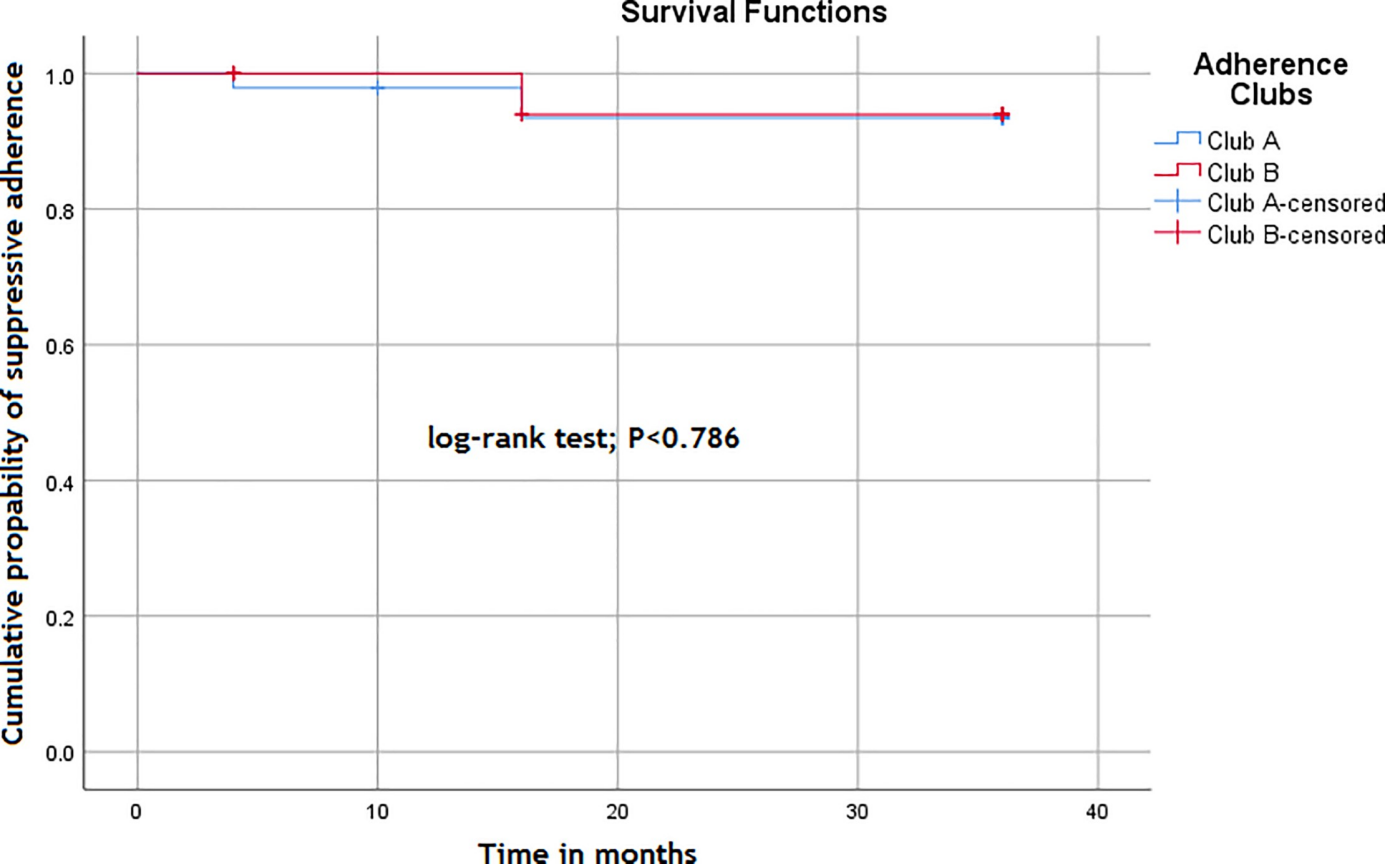
Survival distribution of suppressive adherence behaviour of two adherence clubs at Facility X.

The survival (viral suppression) distributions of patients receiving ART care in the two adherence clubs is displayed in **Fig 5**.

The log-rank test of the survival distributions of the two adherence clubs is 0.786. The lack of statistically significant differences between the survival distributions indicates a consistency in the effectiveness of the adherence clubs in enhancing adherence to medication.

## Qualitative findings

The results of the qualitative studies are presented, based on the framework provided by the two initial programme theories.

### Mechanisms related to initial Programme Theory I

This initial theory relates to how clinically stable patients receiving care in the adherence club programme perceive, interpret and act on the resources and opportunities offered by the club intervention. The mechanisms include perceived social support, perceived benefit, trust, motivation, satisfaction (with care), and self-efficacy.

#### Perceived social support

Perceived social support relates to the feeling of having received or the possibility of receiving moral, emotional and medication-related support from fellow group members of the adherence club [50]. This social support could come in the form of general discussion, informal counselling, or as a response to a question posed by one of the group members. Sometimes, it involves helping a group member to access their medication when they cannot. A patient had this account:

There was a time I had a lot of pimples on my back and I was a bit shy to talk to people but there was another lady here with us in the club, she talked to me openly how she is on this medication, *then* that is when I started to talk to people. They also give ideas; maybe you can use that for the pimples, use that before you go see the doctor if it does not stop, go [and] see the doctor and so on. We talk about it [medication], side effects and so on. ‘How do you feel? Do you feel sleepy when you take your medication? We talk all these things. **[Patient 1]**

We also observed an incident that confirmed the supportive nature of the club members. On one occasion, one of the club members came late, as he had to go to work at a destination (Blouberg) far from the clinic. The patient explained her challenges to the counsellor, who asked her to negotiate with the other club members to get her medication first and leave.

#### Perceived benefit

Perceived benefit relates to the possible advantages that are associated with being in the adherence club (as opposed to being in the clinic care). The benefit relates, in most part, to the tailored and streamlined services offered at the adherence club, which leads to quick service delivery. Aspects that were commonly cited by the participants as beneficial include the quick access to medication and preferential care when needed.

Before it was as if you are sitting here [the main clinic] the whole day in the clinic, here [the ART Unit], they do a very quick job, fantastic job here. However, the problem was always there at the Pharmacy where you go and sit in the queues for the whole day, which means you cannot plan for anything else. Once you come to the clinic, you know you are going to be there the whole day. Now since I am in the club, for me like when I am done here I am going to see the doctor in the next 10 to 15 minutes, after that, I am going back to work. **[Patient 1]**

#### Trust

Trust relates to what extent patients can rely on the health-care providers to provide the support that they need regarding the self-management of their disease. Trust also relates to the integrity of all group members in maintaining a conducive treatment environment. Excerpts from interviews with the various groups of interviewees indicate that the trust that is negotiated between the patient and the health-care providers and fellow group members is an important mechanism of the adherence club intervention.

Patients feel they can talk to each other because they build trust in the group. Because they see each other all the time, they have that trust that they can share information. **[Club facilitator 2]**

#### Motivation

The adherence club intervention constitutes an important source of *extrinsic motivation*. Motivation highlights the use of evolved inner resources for behavioural self-regulation–the process by which people control or alter their thoughts, emotions and behaviours. The evolution of the inner resources could result from the support of others (social motivation) or the prospects of using the resources on offer (outcome expectancies) and the outcome of the previous use of the intervention (result-based motivation) [51]. The adherence club enhances patients’ motivation by 'removing' barriers to access to medication and health-care professionals as revealed in the following excerpts.

The simple fact that they [patients] come here and they pick up their medication, they sit for an hour and a half, two hours, sometimes even less, depending on which type of visit it is, it *motivates* them because sitting in the clinic for one to two hours besides sitting in Pharmacy for two hours waiting period is exhausting. [Longer periods in the clinic] requires patients to take off sick leave, which affects their work. Therefore, with the club, they feel that at least their good adherence and the fact that they [are] looking after themselves, they are rewarded for that as being a club patient. **[Nurse 1]**

The interviews of the study participants revealed that patients who are not yet part of the adherence club intervention are ‘motivated’ to adhere to their medication and remain in care although they are not part of the intervention. This is because these ‘non-club’ patients are told that if they show evidence of good adherence (undetected viral load) and regular clinic attendance, then they qualify to be part of the adherence club. The prospect of becoming part of the adherence club programme motivates the patients to work towards achieving the qualifying goal, which at the same time gets them to adhere to their medication and remain in care. This goal setting and reward system constitute external motivation. This is captured in the following excerpt.

We explain to the patients [the importance of the club]. So, they say to us, ‘I will make sure I am on time. I will make sure I take my tablets. I will make sure my viral load is suppressed. I will show Sister. Next time I will show you.’ That is how the patients become motivated because they know ‘okay there is something I need to work towards to benefit at least from something instead of spending a day at this facility, I can spend half an hour at this facility and off I go.’ **[Nurse 2]**

#### Satisfaction (with care)

Satisfaction relates to the fulfilment of one’s desires, expectations or needs. Satisfaction with ART care received at the club triggers a positive feedback whereby the satisfaction derived from the care received causes increases in their commitment. The patients expressed their satisfaction with the following words.

Everybody in the club is very happy because of the way they [the club team] are working. You do not need to take off when you are working, you come to the club then you go to work when you are done… Their service is very good. **[Patient 2]**

I can say I like it [the club] because you can go to work and there are no long queues, especially as you come and your medication is already there… its very nice man! I am happy with the club; the club makes things easier for us. **[Patient 4]**

#### Knowledge acquisition (learning)

Knowledge acquisition or learning is an important cognitive mechanism identified to perpetuate adherence to medication and retention in care. This could be achieved directly or by enhancing the self-efficacy–the perception that one is capable [has the capacity] of doing or accomplishing a task–of the patient. The excerpts below show how the patients perceived the health talks and counselling received from the adherence club.

Yes, I learn new things. If I am not feeling well, I know what helps me so that the next time when I have the same problem I know what could help me. **[Patient 3]**

It is good to attend those talks because they [patients] pick up false information elsewhere… That one told me I should use that and that…” At the end of the day, it is not suitable for you, for your health. So, we explain to them and they listen to us… **[Club facilitator 1]**

#### Group (identity) dynamics

Group dynamics relates to the cohesion of the group, and this follows from the concept of group identity. If patients identified themselves as part of a group, they would tend to protect the overall interest of the group. To typify the group identity and the relationship group members share, during an interview, one of the participants described how close she is with the other group members and the support she provides based on this relationship.

I know all the people in the club, I know okay ‘this person-that person.’ *For me*, if I do not know some of them personally, at least, I know them by name. We have that relationship ‘okay who are you, where are you from, where you stay and since when are you attending the clinic visits’ and stuff like that. We talk say for instance somebody asks me and says ‘that is my next date I am not going to be in Cape Town, can you get my medication for me and keep it by your house?’ That I will do because I am then a club member and I will go to the nurse and explain to them *with* her and say ‘okay well can I get her medication? I know where she stays; I can take it for her.’ Even if sometimes, say for instance I come here and I see one of the ladies that I always talk to is not here, I am going to phone her or ask her ‘what’s up, what’s wrong, where are you then, I am here, it is our date together.’ I know all the club members; our dates are together… We are always together here on the same date. That is, that is how the relationship is. **[Patient 1]**

While most of the interviewees agreed that there is sharing and support taking place within the groups, our observations revealed a relative lack of explicit interactions between the group members of some clubs, and not always a positive vibe or positive group dynamics within all the clubs. We observed how most of the patients sat quietly, waited for their turn to be called to collect their medication, and to receive preliminary screening. At some point, we noticed two club members engaging in a discussion. We interviewed one of them and asked about their discussion. The discussion was about the possibility of having family members who are not HIV positive to come and learn about the disease to dispel the stigma and discrimination that they display toward HIV-infected family members.

### Mechanisms relevant to initial Programme Theory II

The following mechanisms that were identified related to initial Programme Theory II. This theory relates to the role of the club rules and regulations in propagating retention in care and adherence to medication among patients receiving care in the adherence club programme. This could be seen as the constraints of the adherence club intervention. The main mechanisms identified include perceived threat and being nudged.

#### Perceived threats

In relation to the adherence club intervention, perceived threat refers to an individual’s subjective assessment of the severity of the consequences of non-adherence to ART or to failing to follow the rules of the adherence club. When a patient fails to follow the rules, which is principally about attending all club sessions (or send someone) to collect medication and maintain a lower than detectable viral load, they are returned to the main clinic care. This is what some study participants said about in relation to the club rules.

If your viral load increases, you are out … Therefore, you will rather stay adherent so that the virus is suppressed. If not, you are going to lose out and return to where you started. They do not want to be back into the clinic. Once you mention that, they will rather sacrifice and stay in the club with all the rules–because we are not strict but keep them on track… They need to take responsibility when it is their club date, if they cannot make it, they must communicate. If their viral load is not suppressed, if it increases while they [are] in the club, when we do the blood tests, they will be sent back to the clinic. **[Doctor 1]**

#### Nudging

Being nudged as a mechanism refers to the perception of being stimulated in various ways to make certain choices by setting default options in a specific way. The rules and regulations of the adherence club are meant to guide the patients to attend their club session and adhere to their treatment. This is what the participants think about the club rules and regulations.

If we do not have club rules, you will find that patients will walk in whenever they want. They will not come on their dates and they will not communicate with us. At the end of the day, patients know we do not put them out of the club. They take themselves out of the club by not honouring their appointments and by not looking after themselves because we want them to remain virally suppressed. **[Nurse 2]**

We need to stick to those rules and we need to make sure that they are also taking responsibility; otherwise, they are not going [to] take it as a serious thing. They will think ‘I can do what I want, I can come when I want, nobody checks on me.’ **[Club facilitator 2]**

### Context

Important contextual conditions that were identified are staffing dynamics, collaboration/teamwork, continuity of care, availability of medication, preparation and number of clubs run by the facility.

#### Staffing dynamics

Staffing dynamics relates to the notion of having the desired interdisciplinary members of the adherence club team available to run the club activities. Furthermore, it relates to workload management and having enough time to be involved in the running of the club. The number of staff members available to work in the adherence club programme within the facility and the distribution of these members to the various tasks of the adherence club intervention relate to the context of staffing dynamics. This is what some of the staff members said on staffing dynamics.

You need two people [club facilitators] because one must be focused on the register when they [patients] come in, you write their next appointment date, you must put the weight in the register, you check everything. The other one [club facilitator] is checking the boxes, the medication in the box so that if the medication arrived or if there is no medication so that we can prepare. The doctor can write up medication for that person if their medication did not arrive. Therefore, we need two people, not just one. **[Club facilitator 1]**

#### Collaboration/Teamwork

Teamwork is defined as “a dynamic process involving two or more healthcare professionals with complementary backgrounds and skills, sharing common health goals and exercising concerted physical and mental effort in assessing, planning, or evaluating patient care” [52]. Regarding the adherence care intervention, teamwork relates to the concerted effort of the club team in planning, organisation and execution of the activities of the club sessions. Teamwork influences the actual implementation and running of the adherence club sessions and thus constitutes an important context element for the adherence club intervention.

Teamwork, dedicated team and as you know in the past it used to be Sister A that was running the team with the Counsellors and only now and again when she needs some advice then she will come either to the Doctor or to us as the CNP’s [Clinician Nurse Practitioner]. In addition, she had a very good relationship with the Counsellors; they understand one another. The teamwork was great, they knew exactly which club is coming because there was a system in place that she sort of drafted [specified responsibilities], ‘club this is due for blood tests, this club is due for medication, this club is due for a clinical visit.’ **[Nurse 2]**

Our observation confirmed that the counsellors worked together as a team in both clubs. Although each counsellor has their own clubs s/he facilitates, they come together each day to help each other so that the clubs can be run smoothly and that the patients are served efficiently and as quick as possible. The teamwork extended to the patients themselves. When the patients arrive at their club sessions in the mornings, they find the weighing room open with a scale. They are expected to take their weights and write these down on pieces of paper to be used during the quick-screening process. Encouraging patients to take their weights before the club team arrives fosters the notion of self-management and makes them feel part of the adherence club team rather than just a user.

#### Availability of medication

Quick access to medication is one of the central modalities of the adherence club intervention. Therefore, the availability (or lack) of medication is a critical context condition for the smooth running of the adherence club intervention. The following excerpt shows how important the availability of medication is.

Well, we do not have many challenges but only when the medication does not arrive [from the Chronic Dispensing Unit], then that is the biggest challenge because we have to check the folders, prepare the folders and then run up and down to the Pharmacy. It is very difficult when you are telling the patient ‘your medication is not here’ then the patient knows ‘I am going to sit at [the] Pharmacy’. That is what they do not want. **[Club facilitator 1]**

To demonstrate the importance of receiving one’s medication from the adherence club, one patient recalls the frustrations of a fellow group member who threatened to leave the club because her medication was erroneously processed through the pharmacy rather than the club.

A woman came to the club, but her medication was not here and she had to go to the Pharmacy. Then she said, ‘I think I am going to get out of the club’. I said ‘No, I think maybe you can still carry on. Do not remove yourself from the club, because maybe next time when you come, your medication will be already here.’ So, she was giving up hope because she said it was the second time she was asked to go to the Pharmacy. Then I said ‘No, you can still be a part of the club. Maybe when you come for the third time, it is going [to] be here [at the club]. **[Patient 2]**

#### Preparation

Preparation or the lack of preparation was also identified as an important context element, which sets the stage for how the activities of the club are conducted. The club team agreed that preparation is pertinent to the successful execution of the adherence club sessions.

Like now they are preparing for tomorrow’s club, I think there is a club tomorrow so we went to Pharmacy to check if medication is there so that we know who is going to have medication and who is not going to get so that we can prepare today. Tomorrow morning, when they come we already know who would be going to go to Pharmacy. We start with those people that are going straight to Pharmacy. The doctor does the scripting for the medication so that when patients return for their next visit, their medication is ready. So, we check everything. **[Club facilitator 1]**

**Fig 6:**
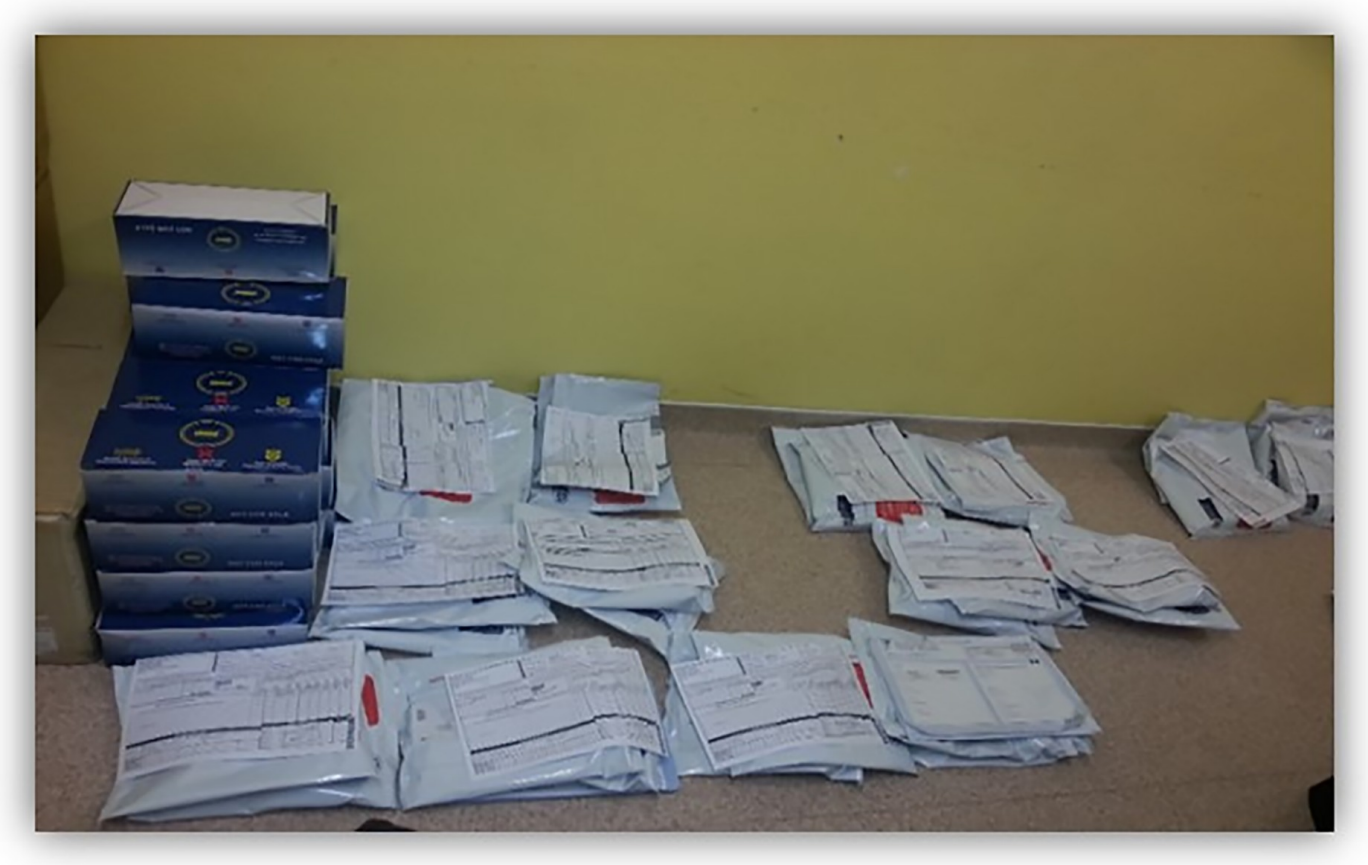
Organised medication packages.

Our observations confirmed that the adherence club team makes sure that they are prepared before each club session. The preparation for the club activities starts the day before, where the counsellors make sure that the folders of the patients have been drawn and that their medication has arrived from the Chronic Dispensing Unit (CDU). Early, on the day of the club, the medication packages are organised, waiting for the patients to collect. See **Fig 6**.

#### Number of clubs run by the facility

The number of clubs that a facility runs is considered an important context factor that influences the running of the intervention, hence, the mechanisms activated by the intervention. This is because the number of clubs that the facility runs affects the scheduling of the number of clubs to be run per day, thus affecting the staffing dynamics and the workload of the club team. These are the opinions of some of the club team members regarding the number of clubs being run at the facility.

Well, it is going to be too much work actually, because we are having lesser clubs compared to other clinics. Other clinics already have about 100, or 150 clubs. So, we are still building up. **[Club facilitator 1]**

Then we have how many clubs now? Fifty-seven I think …Yeah, then each club has about 30 to 40, and others now have in the club 50 patients that we have in them. **[Nurse 1]**

#### The organisation of the facility

The organisation of the facility speaks to the way the HIV-management and Care programme is organised at the facility. Some facilities have ART services as a separate entity from the rest of the clinic whereas others ‘integrate’ ART care services with other chronic non-communicable diseases. Information regarding the organisation of the facility was collected from the structured observations that were conducted. Based on our observation, the MPCHC has organised the adherence club as a unit of its own. This is labelled “Chronic club B” to avoid any stigmatisation of patients seen going to this section of the clinic. The understanding is that patients feel free here and avoid the judging looks of other patients. To this end, they feel no one judges them.

### Outcome

In addition to the two primary outcomes of the adherence club intervention (retention in care and adherence to medication) described using the quantitative methods, other outcomes emerged from the qualitative data. Additional outcomes of the adherence club include reduced workload and decongestion of the facility.

#### Reduced workload

Reduced workload is defined here regarding the number of patients that the health-care providers see per day. The participants suggest that if patients are successfully retained in care, then the workload of the clinicians will be reduced.

The clinic is getting less with patients. So, we do not have to see more than 100 patients a day…At least we can see 50 to 60 a day. Therefore, it helps *us* also as the staff, so that we do not have too much workload. **[Club facilitator 1]**

Definitely, because it looks like now, about plus-minus 1 500 patients are remaining in care *in* the clinic, [who now] are in the club. Which means 1 500 [patients] less that the Clinicians have to see, 1 500 less that [the] Pharmacy has to service except obviously the few who have to go to the Pharmacy [because of scripting errors or whose medication did not arrive]. **[Nurse 1]**

#### Decongestion of the facility

Decongesting the health-care facility is an important health systems outcome, which is also tied to the successful retention of patients in the adherence club system. This is what one of the participants said in this regard.

Decongest, I am thinking in terms of decongesting the Pharmacy area, because of sending, say, 30 to 35 patients to Pharmacy to collect their medication from Pharmacy. They do not need to go to Pharmacy because they get their CDU [Chronic Dispensing Unit] parcels from the clinic…. get their medication and off they go, right? So that is minus 35 patients in the waiting area. **[Doctor 1]**

## Synthesis

The above sections indicate how the interviews and observations yielded evidence in favour of many elements of the initial programme theory. However, realist researchers need to identify patterns or demi-regularities that explain the observed outcomes. This synthesis aims at obtaining conjectured ICAMO configurations. The Kaplan-Meier descriptions (quantitative analysis) allowed us to describe the outcome of retention in care and adherence behaviours. While through the qualitative arm of the study, we identified relevant context elements, important mechanisms and emergent outcomes as they relate to the intervention and actors were identified.

Realist evaluators develop an explanatory understanding of how programmes work on the basis of retroductive inference [53]. In retroduction, the evaluator starts from observed outcomes and identifies and verifies mechanisms that are theorised to have generated the outcomes (**Table 7**).

**Table 7:** Adherence club intervention-context-actor-mechanism-outcome matrix.

Intervention modalities	Context	Actor	Mechanism	Outcome
**Club rules and regulation**	- Standard operating protocol HIV policy	- Patient	- Perceived barriers Perceived threat Nudged	- Adhering to club appointments
**Grouping patients**	- Availability of space for meeting Longevity of patient the club Relationship with other club members	- Patient Group	- Perceived social support Motivation	- Better adherence resulting from developed self-efficacy
**Quick medication access**	- Availability of medication Proper preparation for club session	- Patient	- Perceived benefit Motivation Satisfaction	- Adherence to medication related to medication access
**Prompt continuity of care**	- Availability of clinicians Staffing dynamics Organisation of club activities	- Clinicians Patient	- Trust	- Retained in care through problem resolution
**Club facilitator-patient relationship**	- Staffing dynamics Teamwork/collaboration	- Facilitator Patient	- Trust Perceived support	- Adherence to medication Retention in care
**Overall intervention**	- Buy-in from care providers Preparation and organisation	- Patients Club teams	- Motivation Self-efficacy Satisfaction (with care)	- Improved retention in care and adherence to medication

**Fig 7:**
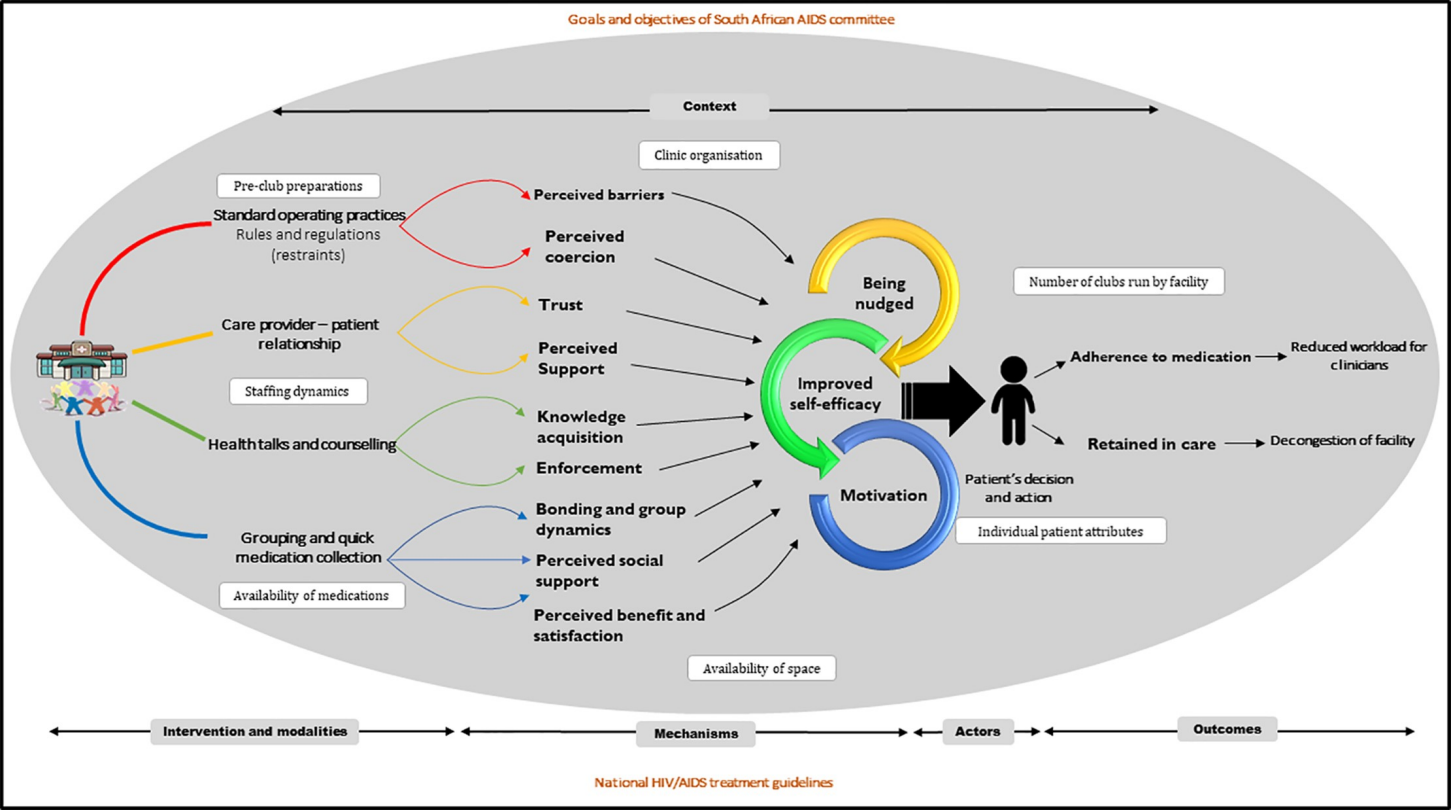
A modified configurational causation model of the adherence club intervention.

In our retroductive inferencing, we applied the ‘configurational’ approach to causality, a logic in which outcomes are considered to follow from the alignment, within a case, of a specific combination of attributes (**Fig 7**) [17]. After obtaining conjectured ICAMO configurations, we applied counterfactual thinking (testing possible alternative explanations) to argue towards *transfactual* (mechanism-centred) conditions [54]. In applying this counterfactual [and transfactual] thinking, we constructed an ICAMO matrix table (**Table 3**).

This ICAMO configuration represents the modified programme theory of the adherence club intervention based on the evidence of the mixed-method approach to data collection and the application of the *retroduction* logic of causal inference. Finally, we used the "if…then…because" statements to translate the ICAMO configuration map into theories (**Box 2**).

**Box 2. Refined programme theory of the adherence intervention**

If adults (18+) clinically stable patients [**Actors**] receiving antiretroviral therapy are grouped for targeted care in which they receive quick uninterrupted supply of antiretroviral medication (with reduced clinic visit frequencies), health talks and counselling, immediate access to a clinician when required and guided by club rules and regulations [**Intervention**] within the context of adequate resources, and convenient (size and position) space and proper preparation by the club team [**Context**], then they feel nudged, their self-efficacy is improved and they become motivated [**Mechanisms**] to continuously adhere to their medication and remain in care [**Outcome**].

The data also indicated that the prospect of joining the adherence club to benefit from its perceived advantages motivates non-members (usually treatment-naïve patients) to remain in regular care and adhere to their medication to meet the criteria for admittance into the adherence club programme.

## Discussion

The aim of the study was to test an initial programme theory of the adherence club in a *real* implementation condition. We sought to confirm, refute and/or modify the initial programme theory of the adherence club intervention by applying a case-study approach and collecting data through multi-methods. Following the process of eliciting the initial programme theory, we identified two possible programme theories (**Box 1**), each offering a different possible explanation of how, why and in what circumstance the adherence club works.

The quantitative findings of this study confirmed those of other quantitative studies [8–11] and, as identified in the initial programme theory that the adherence club intervention enhances adherence to medication and promotes retention in care. A pilot study conducted by Luque-Fenandez and colleagues confirms the good adherence rates of patients using the facility-based adherence club intervention under experimental conditions as obtained in this study [8]. Their findings showed that retention in ART was 94% at 12 months. Another evaluation of the adherence club model under actual implementation conditions also reveals that after 12 months, 95.2% of patients were retained in care [11]. This evidence supports the potential effectiveness of the adherence club in retaining patients in long-term ART care.

Other studies that investigated the effectiveness of the adherence club intervention regarding enhancing adherence to ART showed similar findings as ours [8–11]. Facility-based adherence clubs at 12 months had 2% of patients experience viral rebound (≤400 copies/mL) [8] and the community-based adherence club showed only 1.7% of the patients had viral rebound [9]. In this study, at 36 months, an estimated 4.5% of the patients receiving ART from two adherence clubs had viral rebound. This finding is significant because the global average rate of reporting ≥90% adherence to ART is 62% [55]. These findings confirm the potential effectiveness of the adherence club intervention to enhance population-level adherence to HIV medication.

The non-participant observations and realist interviews employed in this study allowed us to identify intervention modalities, mechanisms and context factors that interact with the mechanism to cause the expected behaviours. Based on the findings of this study, most of the relevant (important) mechanisms that are thought to trigger adherence and retention in care as elicited in the initial programme theory were identified, confirming the theoretical principles of the initial programme theory [27].

Through data obtained from this study, and by applying the transfactual and counterfactual thinking, we uncovered that the two programme theories, rather than being rival theories, complement each other. Thus, the combined (modified) programme theory explains how and why the adherence club enhances adherence to medication and promotes retention in care among stable patients on ART. Most of the respondents indicated that both theories play a role in explicating how and why the adherence club works. Analytically, Hedstrӧm and Swedberg [56] suggest that bundles of identified mechanisms can either work to enhance one another or cancel out each other. Our case study suggests that the bundle of mechanisms enhance each other.

We modified the initial programme theory (**Box 2**) in which case the mechanisms are now considered to complement and enhance each other to explain how and why the adherence club works, based on the findings of our study. However, the extent to which they combine is not exactly clear. In other words, this study did not allow assessing the level of contribution made by each of the theories. We deduce that although these two theories may combine to explain how and why the adherence club intervention works, the degree to which they would combine would depend on the implementation context.

Evidence from other cases have been used to further refine and enhance the programme theory of the adherence club intervention. This is based on the premise that (1) the adherence club intervention could work differently in different settings (as identified in the initial programme theory); (2) the adherence club intervention is also implemented in (slightly) different ways at various facilities; (3) the adherence club intervention may be more effective with some groups rather than others; (4) the adherence club intervention could be more useful in one location than another; and (5) it may have intended and unintended consequences [17]. After obtaining data from other cases, through the process of cross-case analysis, we will seek to elicit a more refined programme theory of the adherence club intervention.

## Rigour and trustworthiness

Several steps were taken to ensure the rigour and trustworthiness of the study. Our sampling process used many criteria to ensure that the information gathered is from a credible source. First, the purposive sampling approach allowed us to select only people who would be information-rich informants. This comprised the health-care providers working directly on the adherence club programme and the patients who were receiving care in the programme.

While seeking for information-rich informants, we also applied the notion of maximum variation sampling. Through this technique, we recruited at least one of each of the cadre working on the adherence club programme. These include the doctors, nurses, club facilitators (counsellors) and the patients themselves. Further to this, we recruited patients of varying duration in the adherence club including those who once were club members and had returned to clinic care or defaulted treatment. Of the five participants included, one of them had been in the adherence club since its inception, while another one had just been in the club for a month. The other three had been in the club within a year’s gap.

To improve the rigour of the study, we applied two levels of triangulation, methods triangulation and triangulation based on the study participants. Adopting the mixed-method approach allowed some aspects of the study to be verified using the other method. Using a variety of participants also promotes the triangulation of the information obtained from them and assists the research to verify facts.

Frequent debriefing sessions were held between the authors. These sessions took place in all the phases of this study including the data collection, analysis and synthesis phases. In conducting and reporting the findings of this study, we followed the RAMESES II reporting standards for realist evaluation developed by Wong and colleagues [57].

## Limitations

The description of the study context is predominantly from the point of views of the health system and health care providers with little inclusion of the patients’ points of view. This misrepresentation constitutes a limitation as it could introduce potential bias. Although we adopted the purposive sampling approach for the qualitative arm of the study, sampling based on who will provide the best information based on pre-existing assumptions is tricky. For this reason, we purposively sampled the participants based on how long they have been part of the adherence club programme. These characteristics are displayed in **Table 2**.

It would have been ideal to investigate the factors influencing survival (retention in club and adherence to medication) based on the personal characteristics of the patients such as age categories, employment status and marital status. However, because each adherence club only has a maximum of 35–45 patients, the results would be misleading. The hazard ratios that would have been obtained would have had wide confidence intervals to make any meaningful inferences.

Studies that require the application of logic in the process of making associations of various elements in a configurational map depend largely on the judgement of the researcher(s). This process, therefore, could be influenced by the line of thinking applied by the researcher(s) to the available data. This is particularly important because it is easy to make causal misattributions given the complexities of the systems we studied and the possibility that different mechanisms can cause the same events. To obtain an objective application of the logical reasoning to the data, frequent debriefing meetings were held by the researchers to judge how best the data fit within the conceptual framework. In addition, the obtained configurational map (programme theory) was presented to middle-level managers who are conversant with how the adherence club works, but are not involved in the evaluation process for their objective scrutiny of the obtained programme theory. The aim was for the managers to judge how well the obtained programme theory represented the actual implementation and running of the intervention.

While this manuscript reports on a ‘high’ performing adherence club case, within the main project, we identified a ‘poor’ performing case to shed more lights on the contextual conditions that could undermine the working of the adherence club intervention. The findings of this ‘control’ case are reported in Mukumbang and colleagues [58]. Findings from these cases studies were used to conduct a cross-case analysis, which informed the refining of the initial programme theory [59,60].

## Conclusion

In this study, we applied the realist evaluation approach to testing the programme theory of the adherence club intervention. Based on the findings of this study, we have made modifications to the initial programme theory, a first step towards refining the initial programme theory of the intervention. Nevertheless, contributions from other case studies are required to provide further confirmations, refutations and modifications of the initial programme theory to obtain a valuable middle range theory. An empirically refined theory has the potential to inform the adaptive programming and implementation of the adherence club intervention in other areas to improve population-level adherence to ART.

## Supporting information

S1 File Observation guide. (DOCX)

S2 File Interview guide–health care providers. (DOCX)

S3 File Interview guide–patients. (DOCX)
