# Supplementary material for: Unravelling how and why the Antiretroviral Adherence Club Intervention works (or not) in a public health facility: A realist explanatory theory-building case study
Source: PLoS One. 2019 Jan 16;14(1):e0210565. doi: 10.1371/journal.pone.0210565 (PMC6334969; doi:10.1371/journal.pone.0210565)
Supplement: S2 File — (DOCX) [file pone.0210565.s002.docx]

**Project:** A realist evaluation of the antiretroviral treatment adherence club programme in selected primary health care facilities in the metropolitan area of Western Cape Province, South Africa:

**Interview guide for clinicians (Medical doctors, club managers, club nurses, club facilitators)**

1. I have heard from some of your colleagues that the adherence club helps patients to take their medication correctly, what in your opinion makes them to take their medication well when they are in the clubs? How do think the adherence club helps them to make that decision to take their medication and continue to attend their clinic appoints?
2. What exactly is it about the adherence club that makes patients to adherence to their medication when they are in the adherence club?
3. Have you experienced for yourself that putting patients in a group helps them to adhere to their medication. How exactly do you think this helps them?
4. Why else do you think that patients in the adherence club will take their medication as instructed and continue to attend their clinic appointments?
5. Why else do you think that patients in the adherence club will take their medication as instructed and continue to attend their clinic appointments?
6. Some people suggest that the patients are being manipulated to attending the adherence club meeting because if they do not attend a meeting they are sent back to the main clinic with long waiting time. Others say that the patients are motivated and empowered to take care of themselves in the club which is why they are adherent. What is your opinion? Which is the case in your experience?
7. Some of your colleagues say that the adherence club motivates the patients to take their medication, in your opinion, in that the case. I so, how is this achieved?
8. It has been suggested that the adherence club empowers the patients. In what ways do you think these patients become empowered to self-manage their disease through the adherence club?
9. When I observed the club meetings, I observe feel like the patients are being controlled towards taking their medication by counting their medications and promising to put them out of the club if they do not attend regularly, is that the case or what is your opinion?
10. I have heard from others that the club helps to decongest the facility, do you feel the same? How this does happens?
11. How do the targets that you receive from the sub-district office affect the way that you run the adherence club? Do you think it affects the way the clubs are being run?
12. Have you experienced for yourself that putting patients in a group helps them to adhere to their medication? If so, how do you think this happens?
13. How does grouping patients together, making their medication readily available and receiving group counselling and education make the patients to adhere to their medication and remain in the care umbrella?
14. I have heard from others that the club helps to decongest the facility, how do you think that is achieved?
15. Why do you think some patients in the adherence club adhere to their medication well and some of them fail to do that?

Thank you for your time and contribution
