# Supplementary material for: Unravelling how and why the Antiretroviral Adherence Club Intervention works (or not) in a public health facility: A realist explanatory theory-building case study
Source: PLoS One. 2019 Jan 16;14(1):e0210565. doi: 10.1371/journal.pone.0210565 (PMC6334969; doi:10.1371/journal.pone.0210565)
Supplement: S3 File — (DOCX) [file pone.0210565.s003.docx]

**Project:** A realist evaluation of the antiretroviral treatment adherence club programme in selected primary health care facilities in the metropolitan area of Western Cape Province, South Africa:

**Interview guide for programme users (adherence club members)**

1. How long have been in the adherence club? Does it help you better to take your medication and attend your clinic appointments?
2. In what ways do you think being in the club helps you to take your medication and always attend your clinic appointments?
3. When you attend the club activities, what makes you to take your medication at the time that you are supposed to take it?
4. Do you interact with the other club members? How does interacting with them help you to take your medication all the time?
5. What do you think about the counselling and education that you receive in the adherence club? Does it really help you to take you mediation? If so how?
6. Do you sometimes receive help from the other members in the club? If so, what kind of help do they offer to you?
7. Do you people share your experiences in the club during the meetings? Have you ever used an advice or a story you have received from a club member to manage your health?
8. Do you people share your experiences in the club during the meetings? Have you ever used an advice or a story you have received from a club member to manage your health?
9. Do you receive any support from the club team members, like the nurses and counsellors? What kind of help do you receive from them? How does that help you with taking your medication?
10. Do you feel like you are being controlled in the adherence club?
11. Do you think it is necessary for the counsellor to do pill counting every time you come to the club? How does that make you feel? What does it make you to think?
12. Have you seen someone sent back to the main clinic? How do you feel or what do you think when they tell you that they will send you to the main clinic if you do not attend all the appointments or send someone to come for your drugs?

Thank you for your time and contribution
